# Risk Factors for Cerebral Palsy in Moldova

**DOI:** 10.3390/medicina57060540

**Published:** 2021-05-28

**Authors:** Ecaterina Bufteac Gincota, Reidun Jahnsen, Larisa Spinei, Guro L. Andersen

**Affiliations:** 1 Department of Health Sciences, Oslo Metropolitan University, P.O. Box 4, St Olavs Plass, 0130 Oslo, Norway; 2 Early Intervention Center ‘Voinicel’, Drumul Taberei Str, Nr 2A, MD-2008 Chisinau, Moldova; 3 Norwegian Quality and Surveillance Registry for Cerebral Palsy (NorCP), Oslo University Hospital, P.O. Box 4956, Nydalen, 0424 Oslo, Norway; reijah@ous-hf.no; 4 Institute of Health and Society, CHARM, University of Oslo, P.O. Box 1130, Blindern, 0318 Oslo, Norway; 5 Department of the Social Medicine and Management, State Medical and Pharmacy University ‘N. Testimitanu’, MD-2004 Chisinau, Moldova; larisa.spinei@usmf.md; 6 Norwegian Quality and Surveillance Registry for Cerebral Palsy (NorCP), Vestfold Hospital Trust, P.O. Box 2168, 3103 Tonsberg, Norway; guro.andersen@siv.no; 7 Department of Clinical and Molecular Medicine, Norwegian University of Science and Technology, P.O. Box 8905, 7491 Trondheim, Norway

**Keywords:** cerebral palsy, Moldova, risk factors, hyperbilirubinemia, asphyxia, prevention, quality registry

## Abstract

*Background and Objectives:* This is the first study assessing risk factors for cerebral palsy (CP) among children born in Moldova. The aim of this study was to identify and describe risk factors for cerebral palsy (CP) among children born in Moldova, which is one of the low-middle income countries in Europe. *Materials and Methods:* We identified 351 children with CP born during 2009 and 2010 in Moldova. Detailed information on 417 children without CP served as a reference group. Logistic regression analyses were applied to the calculate crude and adjusted odds ratios (OR) for CP with 95% confidence intervals (CI) in addition to attributable fraction (AF). *Results:* Among children with CP (40.5% girls), 26% had spastic unilateral, 54% bilateral, 13% dyskinetic, 5% ataxic and 2% unclassified CP. Significant risk factors for CP included maternal alcohol consumption during pregnancy (OR 1.7, *p* = 0.002), maternal hypertension (OR 2.0, *p* < 0.001), children born to mothers from the rural areas (OR 1.6, *p* < 0.001), maternal age ≥35 years (OR 0.6, *p* = 0.018), maternal epilepsy (OR 4.3, *p* < 0.001), breech delivery (OR 3.1, *p* = 0.001), home births (OR 6.3, *p* = 0.001), umbilical cord around neck (OR 2.2, *p* < 0.001), AVD (OR 3.1, *p* < 0.001), male gender (OR 1.3, *p* < 0.001), SGA (OR 1.3, *p* = 0.027), multiple gestations (OR 1.7, *p* < 0.001) and hyperbilirubinemia (OR 4.5, *p* < 0.001). Multivariable analyses showed that the AF of CP was 64% for rural residence (OR 2.8, *p* = 0.002), 87% for home birth (7.6, *p* = 0.005), 79% for pre-labor rupture of membrane (OR 4.9, *p* = 0.001), 66% for breech delivery (OR 2.9, *p* = 0.002) and 81% for hyperbilirubinemia (OR 5.4, *p* < 0.001). *Conclusions:* A combination of factors related to the mother, the delivery and the child were risk factors for CP in Moldova, many of them possibly avoidable. Improved pregnancy and maternity care would potentially reduce the risk of CP. A national CP registry in Moldova is suggested as an opportunity to follow up on these findings.

## 1. Introduction

This is the first study assessing the risk factors for cerebral palsy (CP) among children born in Moldova. The Republic of Moldova is a small country that lies in the central part of the European continent in the north-eastern Balkan, surrounded by the Ukraine on the north, east and south, and separated from Romania on the west by the Prut River.

Moldova is ranked as the country in Europe with the lowest gross domestic product (GDP) per capita, estimated to around USD 5700 in 2017, compared with an average of USD 40,000 for the European Union. These limited financial resources are a likely explanation for the high maternal mortality rate of 23 deaths per 100,000 live births—five to six times higher than in the Nordic countries, and of the infant mortality rate of 12 deaths per 1000 live births. The prevalence of CP and distribution of CP subtypes, motor impairments and associated problems were unknown till 2018 [1].

In Moldova, before 2018, no studies on the CP panorama using the international recommended classifications and evaluations were performed. The only article on CP we succeeded to identify was published in 2011, “Cerebral Palsy—Early Diagnosis” conducted on a cohort of 158 children aged 1 week–24 months old, describing the early signs of CP [2].

The first study on prevalence, subtypes, severity and associated impairments was conducted by the authors of the present article [1]. As a next step we wanted to look into the potential impact on impairments of the early intervention and follow-up programs among children with CP [3].

We consider that the present study represents a starting point to unveil the potential risk factors leading to CP in Moldova. Even though the sample size is relatively small, the accuracy of the data collection results in highlighting the most important potential risk factors and allows future studies to be conducted.

CP is the most common physical disability of childhood, describing a group of permanent disorders of movement and posture attributed to non-progressive disturbances in the developing fetal or infant brain [4]. Potential risk factors have been studied in depth in many developed countries since the 1970s [5,6,7]. Preterm birth (<37 gestational weeks, GW), multiple births, infection in pregnancy, and low birth weight are factors associated with an increased risk for CP [6,7,8].

The present study was performed in Chisinau, Moldova and aimed to identify and describe potential risk factors for CP among children born in Moldova. Data from the birth years 2009–2010 were linked with data from the National Agency for Public Health in Moldova to investigate risk factors for CP.

## 2. Materials and Methods

The recruitment area of the present study covered both rural and urban localities in Moldova. To perform this study, we first conducted a cohort study that allowed us to describe the characteristics of total births for two years (2009–2010): total number of births, neonatal mortality, gestational age, multiple gestations and birth presentation [1].

**Figure 1:**
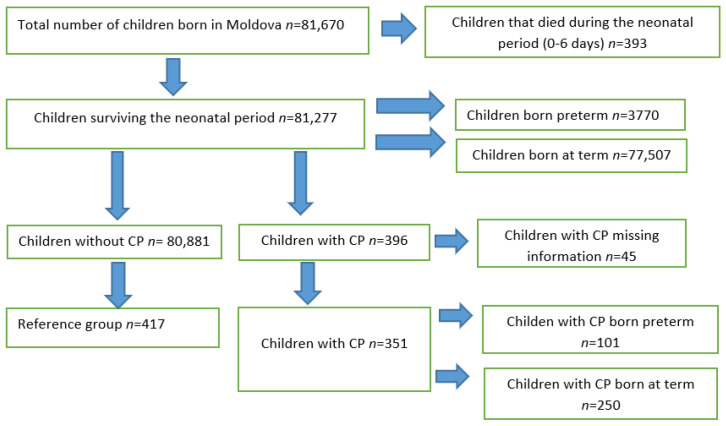
Study population: Children with cerebral palsy (CP) and children without cerebral palsy 2009 and 2010 in Moldova).

Based on these findings, and because no detailed information on other variables was available, a second case-control study was performed, with two groups of children: one with a confirmed diagnosis of CP (*n* = 351) and one without (*n* = 417) (Figure 1).

### 2.1. Children with CP

The group with a confirmed CP diagnosis comprised 351 children born in Moldova between 1st of January 2009 and 31st of December 2010. The CP diagnoses were ascertained by studying the medical records of all children born during the two birth years at the maternity wards, state hospitals for children, orphanages and rehabilitation centers across the country. Each ascertained diagnosis was tracked backwards from the age of seven years, when the diagnosis was confirmed, to the neonatal period, using the medical records from all the relevant departments of the hospitals, such as child neurology, neonatal intensive care units and maternity wards. In this way, we succeeded in covering approximately 95% of the population. In the first article on CP in Moldova from 2018, we described a cohort based on 75% population coverage, with subtypes, severity and prevalence of CP diagnosed in children [1].

Data on maternal health, pregnancy, delivery and the early neonatal period were obtained from the National Agency of Public Health, Chisinau, Moldova [9]. Detailed data on preconception, antenatal and intrapartum risk factors, as well as CP subtypes and the physical and intellectual development of children with CP born during the outlined period, were collected from hospital medical records. The clinical information on CP was collected between 1st of July 2016 and 20th of December 2017, when the children were at least seven years old, with the following inclusion criteria: children (1) born in Moldova, (2) meeting the criteria for CP defined by Surveillance of Cerebral Palsy in Europe (SCPE) [2] and (3) without a genetic diagnosis. Orphanages and habilitation centers in Moldova also provided summary and detailed perinatal and clinical data. The CP subtypes were based on the detailed neurological descriptions in the medical records and in accordance with the SCPE classification [1,5]. Only those children with CP with complete perinatal information were included in the present study.

### 2.2. Reference Group

A cohort of 417 children without CP born in Moldova between the 1st of January 2009 and 31st of December 2010, who survived the early neonatal period (first seven days of life), were included. This group was randomly selected by place of residence and place of birth (rural or urban), to have a reference group that was as representative as possible.

The data were collected from the Institute of Mother and Child and the University Primary Care Clinic, in Chisinau, Moldova, which serve children from all over the country and from the capital, respectively. The data from four general practitioners from the University Primary Care Clinic were randomly selected, with children born during 2009–2010. The main inclusion criteria were the absence of established congenital diagnosis. For the rural area group, the first author randomly selected children born in 2009–2010 from two non-neurological profile departments of the Institute of Mother and Child. In both cases the first author studied the medical records to ensure the correctness and completeness of the data. The clinical information on children from the reference group was collected between 15 May and 30 June 2019.

The Institute of Mother and Child is the only third level hospital for children in Moldova. The children with CP were selected from the neurological departments of the institute, because two times a year all children (from rural and urban areas) with severe neurological diagnoses are referred to this hospital for medical investigations and treatment. The reference group was selected partly from the Institute of Mother and Child (two departments for children aged 4–16 years old) and from the University Clinics. This was the only way to start identifying the children, because all children with severe/unknown neurological diagnosis are referred to this entity from all over the country. The reference group could not be entirely selected from the same hospital “The institute of mother and child”, because this hospital is the only third level hospital for children at the national level, where just mothers with complications during pregnancy and children with severe/unclear diagnosis are referred for special treatment and/or diagnosis clarification. Thus, the controls from the same year of births and places of residence (rural/urban), born both in urban and municipal hospitals, with a different social and educational background and with the same risk of having a baby outside the maternity ward were selected using a combined approach to increase representativeness. None of the clinics have an electronic database for the specified birth years, thus the main researcher studied the available medical records for each child.

### 2.3. Variables

#### 2.3.1. Exposure Variables

Registration of obstetric and perinatal data in the hospital medical records is compulsory in Moldova. All variables were classified into three categories:

##### Mother-Related Variables

Maternal age at delivery was retrieved from the National Agency for Public Health [9] and recorded in three age groups (a: 14–18 years; b: 19–34 years; and c: ≥35 years).

Maternal educational level was recorded as low (primary school), medium (high school) or high (university or college), place of residence as rural or urban, and maternal employment was recorded as yes or no.

Alcohol consumption during pregnancy (>than three units daily) was recorded as yes or no; multiparity (≥3 previous births) as yes or no; socioeconomic status (high, medium and low by the level of income per month of parents); maternal chronic diseases (presence of hypertension, hepatitis, diabetes, intellectual disability, epilepsy and pyelonephritis) as yes or no, were collected from the medical records.

##### Delivery-Related Variables

Mode of delivery was recorded as vaginal birth (vertex or breech) or caesarean section (without specification of planned or emergency delivery).

Pre-labor rupture of membrane (PROM), defined as any rupture of membranes more than 18 h before the onset of labor, meconium waters aspiration, and umbilical cord around neck were recorded as yes or no.

Deliveries outside a maternity ward were defined as home births. Home births also included deliveries at rural general practitioners’ offices with no specialized medical assistance and/or ambulance available.

Assisted vaginal delivery (AVD), also called fundal pressure during the second stage of labor (known as the ‘Kristeller’s maneuver’) involves application of manual pressure to the uppermost part of the uterus directed towards the birth canal, in an attempt to assist spontaneous vaginal birth and avoid prolonged second stage or the need for operative birth. Fundal pressure has also been applied using an inflatable belt. Fundal pressure is widely used; however, methods of its use vary widely. AVD was recorded as yes or no. Instrumental delivery, the use of obstetrical forceps or vacuum extraction, was recorded as yes or no.

##### Child-Related Variables

Gender was defined as boys or girls. Gestational age (GA) in weeks was calculated from the first day of the last menstrual period and classified into term-born (37 weeks or more) or preterm-born (any birth before 37 completed weeks of gestation). Apgar score at five minutes was categorized in three groups 0–3, 4–6 and 7–10. Multiple gestations (more than 1 fetus) were recorded as yes or no. Birth weight (BW) was divided in four categories, <1000 g, 1000–1499 g, 1500–2499 g and >2500 g, and small for gestational age (SGA) was defined as BW below the 10th percentile for the GA [10] and recorded as yes or no.

#### 2.3.2. Outcome Variables

According to the SCPE recommendations, all children diagnosed with CP were classified with spastic, dyskinetic, ataxic or not classified subtypes [5].

### 2.4. Statistical Analyses

Data analyses were performed using SPSS for Windows version 22.0 (SPSS Inc., Chicago, IL, USA). A significance level of 0.05 was chosen.

Descriptive statistics with frequencies, proportions and chi-square tests were used to compare the reference group representativeness and children with and without CP. Odds ratios (ORs) with 95% confidence intervals (CIs) were used to calculate the risk for CP among children using the three categories of variables (child-, delivery- and mother-related). Multivariable logistic regressions were performed to model associations between the outcome (CP) and birth type (multiples and singletons), successively adding covariates GA category and BW categories. The attributable fraction (AF) was calculated to determine the percentage of instances of CP that can be accounted for by a particular risk factor [11].

A variable was considered a confounder if it was associated both with exposure and outcome (CP). Thus, gender, multiple births, GA, maternal age, home birth and SGA were assessed as possible confounders and were corrected for using logistic regression analyses according to the hierarchical model of Victora et al. [11,12]. Singletons born at term were assessed separately for possible confounders, to identify whether they had different risk factors for CP.

## 3. Results

### 3.1. Total Births

During the study period (2009–2010), there were a total of 81,670 births in Moldova. Among the 81,277 (99.5%) children who survived the neonatal period: 3770 (4.6%) were born preterm and 77,507 (95.4%) at term (Figure 1).

Among the 81,277 children, 351 (4.3 per 1000 live births) were diagnosed with CP, and we successfully identified complete information on the variables of interest in 417 children without CP. These children were included as the reference group (Table 1).

### 3.2. Reference Group Representativeness

The reference group was selected from the total population of births for 2009–2010. Due to unavailable databases, the main researcher used two state clinics in order to identify as many children born in the period of interest as possible, both from rural areas (The Institute of Mother and Child) and from urban areas (two university clinics). The above-mentioned clinics have the most detailed information on the children. The two main inclusion characteristics were the place of residence and no congenital diagnoses established. The cases were studied in depth using the paper medical records and all the variables of interest were recorded. The frequency analyses were performed both in the group of total births and in the representative group, using the available data in the total population provided by the National Agency of Public Health [9]. The available data for total births represent the total number of children born in 2009–2010 including those who subsequently died in the neonatal period.

There are no statistically significant differences between the total population and the reference group, in respect of maternal age above 35 years, maternal rural place of residence, breech delivery, home births and child male gender. However, the percentage of premature born children (≤37 GW) and multiple gestations in the reference group was three times higher compared to the total population (*p* = 0.001).

### 3.3. Characteristics of Children with CP Born at Term and Preterm

A particular interest for us was to examine risk factor exposure of children born at term and preterm within the CP group. Thus the proportion of risk factor exposure in children with CP born term and preterm were analysed separately. The results are presented in Table 2.

### 3.4. Characteristics of Children with CP and the Reference Group

Of 351 children diagnosed with CP, 142 (40.5%) were girls, 26% had unilateral, 54% bilateral, 13% dyskinetic, 5% ataxic and 2% had unclassified CP. The proportion of preterm children (29%) was higher among those with CP than in the reference group (17%), OR for CP 1.7, 95% CI 1.2–2.3 (Table 3).

Mother-related variables showed that 251 children (72%) were born to mothers living in rural areas (Table 3), with an overall OR for CP of 1.6 and 95% CI 1.3–2.1 (Table 3). Other risk factors for CP were maternal age ≥35 years (OR 0.6, 95% CI 0.4–0.9), alcohol consumption during pregnancy (OR 1.7, 95% CI 1.2–2.6), and chronic diseases, such as hypertension (OR 2.0, 95% CI 1.3–3.0) and epilepsy (OR 4.3, 95% CI 1.6–11.6) (Table 3).

After multivariable analysis of mother-related variables, maternal age ≥35 years (OR 0.6, 95% CI 0.4–0.9) and rural residence (OR 2.8, 95% CI 1.9–4.1) remained statistically significant. The AF for rural residence and CP was 64% (Table 4).

Delivery related factors that increased the risk for CP in all children were AVD (OR 3.1, 95% CI 1.5–6.9), home birth (OR 6.3, 95% CI 1.8–21.9) and umbilical cord around the neck (OR 2.2, 95% CI 1.7–3.1) (Table 3).

Multivariable analyses showed that the AF was 87% for home birth, 79% for PROM and 66% for breech delivery (Table 4).

The child-related risk factors for CP were: male gender (OR 1.3 95% CI 1.1–1.6, prematurity with OR 1.7 and 95% CI 1.2–2.3, SGA with OR 1.3 and 95% CI 1.1–1.8, multiple gestations, with OR 1.7 95% CI 1.0–2.7 and hyperbilirubinemia with OR 4.5 and 95% CI 2.9–6.9 (Table 3).

Hyperbilirubinemia and Apgar score at five minutes (0–3) had 84% AF of CP and multiple gestations almost 75%.

In multiple logistic regression analyses in all the children, only male gender and hyperbilirubinemia were associated with increased risk of CP. The AF for hyperbilirubinemia and CP was 81% (Table 4).

In relation to multiple births, we found that multiples born moderately preterm (32–36 GW) had a higher risk of CP compared to singletons born preterm OR 0.404 95% CI 0.181–0.905, *p* = 0.028. Moreover, children from multiple births with a moderate low BW (1500–2499g) had a 30% increased risk of CP in comparison to singletons with the same BW (OR 0.337 95% CI 0.124–0.905, *p* = 0.033). Children born severely preterm and with extremely low BW had no statistically significant difference for CP compared to singletons from the same gestational age and birth weight groups.

Table 5 shows that among the 217 singletons born at term with CP, 149 (69%) were from rural areas, in comparison to the 133 (42%) in the reference group.

### 3.5. Characteristics of Singletons Born at Term with and without CP

Among singletons born at term, the highest risk for CP was associated with maternal hypertension (OR 1.9, 95% CI 1.2–3.2), epilepsy (OR 5.1, 95% CI 1.6–15.6), age ≥35 years (OR 0.6 95% CI 0.3–0.9) and alcohol consumption during pregnancy (OR 1.9, 95% CI 1.2–3.2) (Table 5). Multiparity doubled the risk of CP (OR 1.8, 95% CI 1.1–2.9). The AF for maternal epilepsy and CP was 81%, for maternal hypertension and CP 54% and for multiparity and CP 47%.

Among delivery related risk factors for CP in singletons born at term, breech presentation (OR 5.3, 95% CI 2.5–11.3), AVD (OR 3.1, 95% CI 1.3–7.3, home births (OR 3.9 95% CI 1.0–14.7 and umbilical cord around neck (OR 2.2 95% CI 1.4–3.3) showed an increased risk of CP. The AF of CP was 83% for breech delivery, 75% for home births, 69% for AVD and 63% for umbilical cord around the neck (Table 5).

Child-related risk factors in singletons born at term were male gender with an OR 1.5 95% CI 1.1–1.9 (AF = 39%) and hyperbilirubinemia OR 4.0 95% CI 2.4–6.4 (AF = 81%) (Table 5).

The multivariable analyses highlighted the following risk factors among singletons born at term: rural residence (OR 3.4, 95% CI 2.1–5.6); maternal age ≥35 years (OR 0.6, 95% CI 0.3–0.9); male gender (OR 1.6, 95% CI 1.3–2.0) and SGA (OR 3.9, 95% CI 1.5–10.2), and hyperbilirubinemia (OR 0.327, 95% CI 0.159–0.672). The hierarchical split of AF of CP was 74% for SGA, 70% for rural residence and 39% for male gender (Table 6).

## 4. Discussion

In this first study on risk factors for CP in Moldova the highest risk of CP was found among children with potential traumatic events during delivery and hyperbilirubinemia during the neonatal period. Maternal residence in rural areas, low socioeconomic status, chronic diseases and alcohol consumption during pregnancy all increased the risk of CP and had high AF. Home birth and breech delivery, hyperbilirubinemia, Apgar score (0–6) at five minutes, and multiple gestation, also had high AF of CP. These risk factors were also significant among singletons born at term. AVD and instrumental deliveries had the highest AF for CP, although not significant when adjusted for GA, home-births and hyperbilirubinemia.

### 4.1. Strengths and Limitations

A strength of the study is that in all 351 children with CP, the diagnosis was confirmed based on the medical records and physical examination of the children by the age of seven years. This makes it less likely that the CP diagnosis was incorrect. The SCPE recommends that the diagnosis is confirmed by the age of 5 years to avoid misclassification. The CP subtypes were also classified in accordance with the SCPE recommendations. The registration of risk factors was done prospectively making recall bias less likely.

A limitation of our study is the small cohort in comparison with other, larger studies [13,14,15,16,17,18,19,20,21]. Another limitation is that the study is not completely population-based. We did, however, manage to include approximately 95% of the population of children with CP and therefore consider that the findings are representative for the situation in Moldova.

The reference group we succeeded to select from the two state hospitals with the most detailed available data, came to be representative for the two years of births in Moldova (2009–2010). The percentages of maternal age and rural residence, breech delivery, home births as well as male gender were comparable in both groups. However, the reference group showed a higher percentage of preterm born babies and multiple gestations, thus, these results should be interpreted with caution, since both these factors are known risk factors for CP [13,14,19,20,21]. The CI’s, for some risk factors, were large (e.g., instrumental delivery, Apgar score); therefore, these results should be interpreted with caution. However, the statistical significance strengthens the credibility of the results.

### 4.2. Comparison with Other Studies

Our first study from 2018, on the prevalence and severity of CP in Moldova in comparison to high income countries in Europe, showed an almost two-fold increase in the number of children with severe forms of CP and associated impairments [1]. The present manuscript is a follow-up study to examine the potential risk factors for CP from three presented groups of variables.

Regarding mother-related variables, our study showed that maternal age over 35 years had a higher risk of having a baby with CP. This might be explained both by chronic diseases at older ages, including hypertension, hepatitis and pyelonephritis as described, and by higher parity. However, this effect can be prevented if women receive quality prenatal care and deliver in a healthcare facility well-equipped to care for high-risk pregnancies and neonates. We also found that this category of women had low socioeconomic status, which might indicate poor nutrition and low access to quality medical care. Other studies have also concluded a higher risk of a child with CP among women over 30 years old [22]. The results from a Chinese study (2017) indicated 20 significant risk factors for CP, among which the most important were maternal age >30 years, maternal alcohol consumption during pregnancy, rural residence, paternal occupational exposure to harmful sub-stances, and multiple births. This study concluded that maternal habits during pregnancy and rural residence had an important role and that the CP incidence could probably be further lowered if these factors were considered [22]. However, some studies show that maternal age below 20 years is also associated with an increased risk of CP [23].

Multivariable analyses revealed that rural residence and maternal age above 35 years increased the risk of having a baby with CP. Parturient women who had gestational hypertension and other diseases, such as pyelonephritis, hepatitis and epilepsy also had an increased risk of children with CP, probably due to premature birth, low BW, fetal distress, SGA infants and other abnormal perinatal outcomes. These risk factors for CP were identified in a 2011 systematic review by Himmelmann et al. [20].

In Moldova, because particular attention is paid to non-communicable diseases (NCD), the largest causes of morbidity and mortality in the local population, these diseases are recorded in detail for parturient women. The Millennium Goals established by the World Health Organization for the end of 2020 in Moldova are to reduce the morbidity of NCD in the population. A more detailed, population-based study is required to identify potential harms to the fetus during the perinatal period [24].

Potential traumatic events during labor were found to be important delivery-related risk factors for CP among children born in Moldova. We found that children born in breech delivery, after instrumental delivery or in AVD had a greatly increased risk of developing CP. These results are in line with a study performed in Sweden in 2006, where the authors concluded that several obstetric factors and low Apgar scores were associated with CP [16]. Our results are also in line with the findings from developing countries. A study from Botswana in 2017 showed that significant risk factors for CP included complications during delivery. The study concluded that the major risk factors for CP in Botswana differ from those described in high-resource settings [25].

A Cochrane review on fundal pressure during the second stage of delivery showed that the existing publications did not prove the importance of these maneuvers in shortening labor or reducing harmful impacts on the health status of the mother and child, such as fetal distress, failure to progress, and maternal exhaustion [26]. Our study showed that the AVD and instrumental delivery is an important risk factor for developing CP. These results are partially in line with a population-based cohort study from 1999 to 2010, performed in 2014 in Sweden. The authors concluded that vacuum-assisted delivery was associated with an increased risk of neonatal intracranial hemorrhage compared to Caesarean section [27]. However, when we adjusted for other risk factors, instrumental deliveries and AVD among children born in Moldova were not statistically significant.

Additionally, the instrumental delivery had a very wide 95% CI, thus a more in-depth study should be performed, using a larger group of children diagnosed with CP and a larger reference group. Vacuum extraction and forceps are also frequently used in Moldova, so further population-based studies are crucial.

Our study identified that home births were an important risk factor associated with insufficient perinatal care for both mothers and newborns. These results are in line with a study from China where the authors concluded that births at the hospitals were significantly associated with reduced risk of CP [22]. Home deliveries are likely to have higher risks because they are mostly performed by untrained birth attendants. Rural residence and home births were found more commonly in the group with CP in our study. Low levels of births at hospitals have also been found to be related to CP in other studies from China and the USA [17,22].

We found that breech delivery was a significant risk factor for CP in Moldova, particularly among singletons born at term (≥37 GW). The same results were obtained in a study from Sweden in 2006 and in the 2009 Norwegian study by Andersen et al. [16,17]. However, in our study, breech delivery was not described in correlation with other variables because of insufficient available data. Thus, a more detailed study is required in Moldova.

The univariable analyses showed that PROM >18 h was found to have a 20% increased risk for CP among singletons born at term in Moldova. This finding may suggest a poor delivery protocol in these cases. A national registry cohort study in Norway on term-born singletons from 1999–2009 concluded that intervals of more than 24 hours between PROM and delivery were associated with CP, but not with neonatal mortality or death during delivery [19].

In respect of child-related factors, our study showed a lower proportion of prematurely born children with CP compared to a Norwegian study, which could be explained by a different structure of risk factors for CP in Moldova, i.e., intra- and post-natal risk factors among children born at term constitute a large proportion of the CP population [21].

Another explanation of the low incidence of CP in Moldova among preterm children could be the high rate of neonatal deaths in Eastern Europe. According to a UNICEF report, over the last three decades (1990–2017), neonatal mortality has been decreasing in industrial developed countries in Europe. However, in Eastern Europe, neonatal mortality was three times higher than in western countries in 2019, at 153.240 versus 30.804, respectively [28]. In Moldova, in 2009–2010, the birth years of this study population, the neonatal mortality index was estimated to be 14.8, six times higher than the Norwegian neonatal mortality index (2.6) in the same period [28].

The largest proportion of total neonatal deaths is due to extremely premature babies, as shown in the results of a local study by Paladi et al. [29]. The study included 863 babies born with extremely low BW (500–1000 g) between the 22nd and 28th gestational weeks during 2008–2012. The authors determined a 3–4 times lower survival of these children compared to those in perinatal centers in high income countries. Due to Ministry of Health perinatology strategies implemented in recent years, neonatal mortality has decreased from 100.2‰ to 56.6‰ [29].

Our results are in line with a systematic review from 2013 on risk factors for CP among children born at term in high-income countries [14]. The authors found that 10 risk factors were statistically significant in each study: placental abnormalities, major and minor birth defects, low birth weight, meconium aspiration, emergency caesarean section or instrumental delivery, birth asphyxia, neonatal seizures, respiratory distress syndrome, hypoglycemia and neonatal infections. Instrumental deliveries (compared with spontaneous vaginal or elective caesarean deliveries) were associated with increased risk of CP, as was breech delivery. However, the study concluded that there is a need for more in-depth investigation of the associations [14].

The proportion of children born prematurely or SGA among those who developed CP in our study was significantly higher compared to the reference group, which is in line with a study from the Australian Collaborative Cerebral Palsy Research Group in 2011 including 587 individuals with CP and a large control group. The study reported that preterm birth and intrauterine growth retardation were the largest risk factors for CP [13]. These results are also comparable with a systematic review conducted in 2016 by Pascal Van Liesholt et al. [30]. The included studies indicated that lower gestational age, maternal medical diagnoses, preeclampsia, low birth weight, and the combination of male gender with preterm birth or low birth weight were found to be positively associated with CP [30].

We also found that children born with a low or extremely low Apgar score at five minutes had a six-fold increased risk of developing CP, these results are in line with a registry-based Norwegian study [21].

An important finding related to the child was hyperbilirubinemia, which was found to be six times higher among children with CP than in the reference group. This finding is in line with a study conducted in the USA on the total of children born at term from 1995–2011. The authors concluded that CP consistent with kernicterus occurred with an incidence of 0.57 per 100,000 births; all had high total serum bilirubin levels and at least two risk factors for neurotoxicity, such as prematurity, glucose-6-phosphate dehydrogenase deficiency or hypoxia-ischemia [15].

Multivariable logistic regression showed that multiples born moderately preterm or with a moderately low BW had a higher risk of CP, but multiples born severely premature or with extremely low BW had no statistically significant difference for CP compared to singletons. These findings are in line with the findings from a recent population-based cohort study, which included 12 CP registers across Europe [31]. The authors concluded that CP prevalence among multiples born less than 32 gestational weeks or with BW < 1500 g declined significantly since 1990, becoming comparable to the CP prevalence among singletons of the same GA [31].

The restricted analyses on singletons born at term in Moldova showed the same variables as causes for CP, with a higher risk among boys, children born of mothers from rural areas, children born of mothers aged ≥35 years, children with SGA and children who had experienced hyperbilirubinemia. Following the multivariable regression, the OR for maternal hypertension became statistically insignificant. These results are comparable with several other international studies both from high- and low-income countries [14,15,16,17,18,19,20,21,22,23].

## 5. Conclusions

In this study, we found that a combination of factors related to the mother, the delivery process and the child were risk factors for CP among children born in Moldova. Our findings suggest that improved perinatal care, following protocols in PROM and other potential hypoxic events, better follow-up during pregnancy, avoiding harmful habits and encouraging birth at maternity wards would potentially reduce the risk of CP. Detailed data on chronic maternal diseases, alcohol consumption and smoking should be reported by maternities to the Agency of Public Health. Further studies with larger numbers of participants are required in Moldova to be able to draw conclusions at a population level. A national CP registry in Moldova, linked to the National Agency for Public Health Data, could be a way to follow up our findings further and to monitor the quality of pregnancy, delivery and neonatal care, in addition to enable studies at a national level.

## Figures and Tables

**Table 1:** Reference group representativeness.

Variables	Total Number of Children Born in Moldova 2009–2010 (*n* = 81,670) *n* (%)	Reference Group 2009–2010 (*n* = 417) *n* (%)	*p*-Value
**Maternal factors**			
Maternal Age			
<18 years old	4988 (6)	26 (6)	1.0
>35 years old	10,611 (13)	66 (16)	0.069
Place of residence (rural)	32,904 (41)	184 (44)	0.21
**Delivery factors**			
Breech delivery	2402 (3)	19 (4)	0.23
Home births ^a^	680 (0.9)	3 (0.7)	0.66
**Child related factors**			
Gender (male)	41,807 (50)	192 (46)	0.1
Gestational age <37 weeks	3770 (5)	72 (16)	0.001
Multiple gestations ^b^	1694 (2)	30 (7)	0.0001

^a^ Homebirths-deliveries outside a maternity ward; ^b^ multiple gestations—more than 1 fetus.

**Table 2:** Multivariable analyses with confounders of risk factors among term and preterm born children with CP. (*n*= 351, *n* = 250 children born at term (≥37 gestational weeks), *n* = 101 children born preterm (<37gestational weeks).

	Variables	*n* (%)	Odds Ratio	Lower 95% CI	Upper 95% CI
**Children born at term**	**Maternal factors**				
	Maternal age (>35 years old) Mother chronic diseases	25 (10)	1.8	1.1	3.1
	-Hypertension	52 (21)	3.4	1.7	7.1
	-Pyelonephritis	72 (29)	0.02	0.01	0.3
	Place of residence (rural)	174 (70)	0.3	0.2	0.4
	**Delivery factors**				
	Breech delivery	37 (15)	4.9	1.9	12.8
	Home births ^a^	11 (4)	0.06	0.01	0.4
	Meconium water aspiration	89 (36)	47.3	4.6	83.6
	Umbilical cord around neck	77 (31)	7.3	2.8	19.1
	**Child related factors**				
	Gender (male)	153 (61)	1.7	1.06	2.6
	Birth weight 1500–2499 g	19 (8)	0.15	0.04	0.5
	Small for gestational age ^b^	33 (13)	4.7	1.8	11.8
	Hyperbilirubinemia	83 (33)	2.9	1.5	5.9
**Children born preterm**	**Maternal factors**				
	Maternal age (>35 years old) Mother chronic diseases	10 (10)	4.1	1.1	16.4
	-Hypertension	19 (19)	4.4	0.9	21.6
	-Pyelonephritis	28 (28)	8.3	0.5	19.2
	Place of residence (rural)	77 (76)	0.22	0.07	0.7
	**Delivery factors**				
	Breech delivery	13 (13)	1.1	0.2	5.01
	Home births ^a^	5 (5)	0.9	0.2	1.3
	Meconium waters aspiration	32 (32)	3.4	0.3	20.3
	Umbilical cord around neck	33 (33)	0.9	0.11	15.4
	**Child related factors**				
	Gender (male)	56 (55)	0.5	0.2	1.4
	Birth weight 1500–2499 g	26 (26)	2.3	0.3	19.3
	Small for gestational age ^b^	63 (62)	0.5	0.2	1.5
	Hyperbilirubinemia	35 (35)	6.1	1.3	29.1

^a^ Homebirths-deliveries outside a maternity ward; ^b^ small for gestational age–birth weight below the 10th percentile for the gestational age.

**Table 3:** The mother-, delivery- and child-characteristics in children with cerebral palsy (CP) and the reference group.

	Children with CP *n* = 351 (%)	Reference Group *n* = 417 (%)	Unadjusted Odds Ratio	Lower 95% CI	Upper 95% CI
**Maternal Factors**					
Multiparity ^a^	45 (13)	15 (4)	3.6	1.9	6.5
Maternal Age					
<18 years old	23 (7)	26 (6)			
19–34 years old	293 (83)	325 (78)			
>35 years old	35 (10)	66 (16)	0.6	0.4	0.9
Mother education level ^b^	77 (22)	71 (17)	1.3	0.9	1.9
Alcohol consumption ^c^	67 (19)	46 (11)	1.7	1.2	2.6
Place of residence (rural)	251 (72)	184 (44)	1.6	1.3	2.1
Hypertension	71 (20)	42 (10)	2.0	1.3	3.0
Hepatitis	27 (8)	18 (4)	1.8	1.0	3.3
Pyelonephritis	100 (29)	120 (28)	0.9	0.7	1.4
Diabetes	40 (11)	35 (8)	1.4	0.8	2.2
Intellectual disability	20 (6)	12 (3)	2.0	0.9	4.1
Epilepsy	18 (5)	5 (1)	4.3	1.6	11.6
Low social-economic status	71 (17)	63 (15)	1.3	0.9	1.9
**Delivery factors**					
Breech delivery	50 (14)	19 (5)	3.1	1.8	5.4
C-section	84 (24)	99 (23)	1.1	0.8	1.5
Assisted Vaginal Delivery ^d^	24 (3)	9 (1)	3.1	1.5	6.9
Instrumental delivery ^e^	65 (9)	2 (0.3)	38.6	9.4	158.8
Home births ^f^	16 (5)	3 (1)	6.3	1.8	21.9
Premature Rupture Membranes					
<12 h	261 (74)	347 (83)			
12–18 h	47 (13)	60 (14)			
>18 h	43 (13)	10 (3)	5.1	2.5	10.3
Meconium waters aspiration	121 (35)	120 (28)	1.2	0.9	1.6
Umbilical Cord around neck	108 (31)	58 (14)	2.2	1.7	3.1
**Child related factors**					
Gender (male)	209 (59)	192 (46)	1.3	1.1	1.6
Gestational age <37 weeks	101 (29)	72 (17)	1.7	1.2	2.3
Birth weight <1000 g	12 (3)	9 (2)	1.6	0.7	3.8
1000–1499 g	27 (8)	16 (4)	2.0	1.1	3.8
1500–2499 g	71 (20)	53 (13)	1.6	1.0	2.3
>2500 g	241 (69)	339 (81)	0.8	0.7	1.1
**Apgar score at 5 min**					
0–3	20 (6)	4 (1)	5.9	2.0	17.5
4–6	79 (22)	77 (18)	1.2	0.9	1.7
7–10	252 (72)	336 (81)			
Small for gestational age ^g^	96 (27)	88 (21)	1.3	1.0	1.8
Multiple gestation ^h^	42 (12)	30 (7)	1.7	1.0	2.7
Hyperbilirubinemia >1 week	118 (34)	31 (7)	4.5	2.9	6.9

^a^ Multiparity—≥3 previous babies; ^b^ mother low education—primary school; ^c^ alcohol consumption during pregnancy (>than three units daily); ^d^ assisted vaginal delivery—Kristeller’s maneuver, belt; ^e^ instrumental delivery—vacuum, forceps; ^f^ homebirths—deliveries outside a maternity ward, ^g^ small for gestational age—birth weight below the 10th percentile for the gestational age; ^h^ multiple pregnancies—more than 1 fetus.

**Table 4:** Multivariable analyses ^a^ of the mother, delivery and child risk factors for cerebral palsy (CP), *n* = 351.

	Odds Ratio	95% CI		AF ^b^ %
		Lower	Upper	
**Maternal Factors**				
Place of residence				
Rural	2.8	1.9	4.1	64
>35 years old	0.6	0.4	0.9	
Mother chronic diseases				
-Hypertension	0.2	0.1	0.5	
**Delivery factors**				
Breech delivery	2.9	1.5	5.9	66
Home birth	7.6	1.9	31.3	87
Premature Rupture of Membranes				
>18 h	4.9	1.9	12.2	79
Umbilical Cord around neck	0.3	0.2	0.5	
**Child related factors**				
Gender (male)	0.7	0.5	0.9	
Hyperbilirubinemia	5.4	3.1	9.4	81

^a^ Confounders—gender, multiple births, GA, maternal age, home birth and SGA; ^b^ AF—the proportion of babies with the exposure that may have been caused by it %.

**Table 5:** Characteristics of singletons at term with cerebral palsy (CP) compared to the reference group.

	Children with CP *n* = 217 (%)	Reference Group *n* = 314 (%)	Unadjusted Odds Ratio	Lower 95% CI	Upper 95% CI
**Maternal Factors**					
Multiparity ^a^	27 (12)	22 (7)	1.8	1	3.2
Maternal age					
<18 years old	16 (7)	22 (6)			
19–34 years old	180 (83)	269 (78)	0.9	0.7	1.3
>35 years old	21 (10)	54 (16)	0.6	0.3	0.9
Maternal low education ^b^	44 (20)	53 (17)	1.2	0.8	1.9
Alcohol consumption ^c^	42 (19)	34 (11)	1.8	1.1	2.9
Place of residence rural	149 (69)	133 (42)	1.6	1.2	2.2
**Maternal chronic diseases**					
-Hypertension	39 (18)	29 (9)	1.9	1.2	3.2
-Hepatitis	16 (7)	13 (4)	1.8	0.8	3.8
-Pyelonephritis	60 (28)	86 (27)	1	0.7	1.5
-Diabetes	28 (13)	30 (10)	1.4	0.8	2.3
-Intellectual Disability	10 (5)	11 (3)	1.3	0.6	3.2
-Epilepsy	14 (7)	4 (1)	5.1	1.6	15.6
Low social-economic status	72 (33)	104 (33)	1	0.7	1.4
**Delivery factors**					
Breech delivery	33 (15)	9 (3)	5.3	2.5	11.3
C-section	52 (24)	62 (20)	1.2	0.8	1.8
Assisted Vaginal Delivery ^d^	17 (8)	8 (2)	3.1	1.3	7.3
Instrumental delivery ^e^	39 (18)	2 (1)	28.2	6.7	118.1
Home births ^f^	8 (4)	3 (1)	3.9	1	14.7
Premature Rupture of Membranes					
>18 h	31 (14)	8 (3)	5.6	2.5	12.4
Meconium waters	76 (35)	87 (28)	1.3	0.9	1.8
Umbilical Cord around neck	68 (31)	45 (14)	2.2	1.4	3.3
**Child related factors**					
Gender (male)	139 (64)	137 (44)	1.5	1.1	1.9
Apgar score at 5 min					
0–3	11 (5)	0 (0)	33.3	1.9	567.4
4–6	33 (15)	32 (10)	1.5	0.9	2.5
7–10	173 (80)	282 (90)	0.9	0.7	1.1
Small for gestational age ^g^	32 (15)	49 (16)	0.9	0.6	1.5
Hyperbilirubinemia >1 week	71 (33)	26 (8)	4	2.4	6.4

^a^ Multiparity—≥3 previous babies; ^b^ mother low education—primary school; ^c^ alcohol consumption during pregnancy (>than three units daily); ^d^ assisted vaginal delivery—Kristeller’s maneuver, belt; ^e^ instrumental delivery—vacuum, forceps; ^f^ homebirths—deliveries outside a maternity ward, ^g^ small for gestational age—birth weight below the 10th percentile for the gestational age; ^h^ multiple pregnancies—more than 1 fetus.

**Table 6:** Multivariable analyses ^a^ of risk factors for cerebral palsy (CP) in singletons born at term.

	Odds Ratio	95% CI		AF ^e^ %
		Lower	Upper	
**Maternal Factors**				
Rural	3.4	2.1	5.6	70
>35 years old	0.6	0.3	0.9	
Mother chronic diseases				
-Hypertension	0.2	0.1	0.5	
Multiparity	0.4	0.2	0.8	
**Delivery factors**				
Breech delivery	0.2	0.09	0.6	
Meconium waters aspiration	0.02	0.002	0.2	
Premature Rupture of Membranes				
>18 h	0.3	0.1	0.9	
Umbilical Cord around neck	0.1	0.03	0.2	
Assisted Vaginal Delivery ^c^	1.2	0.4	3.1	
Instrumental Delivery ^d^	1.9	0.9	4.1	
**Child related factors**				
Gender (male)	1.6	1.3	2	39
Hyperbilirubinemia	0.3	0.2	0.7	
Apgar at 5 min (0–3)	1.9	0.9	3.8	
Small for gestational age ^b^	3.9	1.5	10.2	74

^a^ Confounders—gender, maternal age, home birth and SGA; ^b^ small for gestational age—birth weight below the 10th percentile for the gestational age; ^c^ assisted vaginal delivery—Kristeller’s maneuver, belt; ^d^ instrumental delivery—vacuum, forceps; ^e^ AF—the proportion of babies with the exposure that may have been caused by it.

## Data Availability

The datasets generated and analysed in this study are not publicly available due to restrictions regarding the sharing of data with third parties. The Management Committee of the Institute of Mother and Child, the hospital that provided the data, has specified that it is forbidden to share the data with third parties. Data are available from the corresponding author upon reasonable request.
